# Acculturation and Cardiovascular Risk Screening among African Immigrants: The African Immigrant Health Study

**DOI:** 10.3390/ijerph19052556

**Published:** 2022-02-23

**Authors:** Oluwabunmi Ogungbe, Ruth-Alma Turkson-Ocran, Binu Koirala, Samuel Byiringiro, Xiaoyue Liu, Sabrina Elias, Danielle Mensah, Emmanuel Turkson-Ocran, Manka Nkimbeng, Joycelyn Cudjoe, Diana Baptiste, Yvonne Commodore-Mensah

**Affiliations:** 1 Johns Hopkins University School of Nursing, Baltimore, MD 21205, USA; bkoiral1@jhu.edu (B.K.); sbyirin1@jh.edu (S.B.); xliu183@jhmi.edu (X.L.); sdesouz5@jhu.edu (S.E.); dbaptis1@jhu.edu (D.B.); ycommod1@jh.edu (Y.C.-M.); 2 Beth Israel Deaconess Medical Center, Division of General Medicine, Section for Research, Boston, MA 02215, USA; rturkson@bidmc.harvard.edu; 3 Drexel University College of Medicine, Philadelphia, PA 19129, USA; dm3546@drexel.edu; 4 Greater Accra Regional Hospital, Accra, Ghana; ecntocran@gmail.com; 5 School of Public Health, University of Minnesota, Minneapolis, MN 55455, USA; manka@umn.edu; 6 Inova Fairfax Hospital, Falls Church, VA 22042, USA; joycelyn.cudjoe@gmail.com; 7 Johns Hopkins Bloomberg School of Public Health, Baltimore, MD 21205, USA

**Keywords:** aculturation, culture, African immigrants, cardiovascular, hypertension, dyslipidemia

## Abstract

Acculturation and immigration-related factors may impact preventive, routine cardiovascular risk (CV) screening among African immigrants. We examined the associations between length of stay, percent of life spent in the U.S. (proxy for acculturation), and CV screening. Outcomes were recent screening for hypertension, diabetes, and dyslipidemia. Multivariable logistic regression analyses were used to examine these relationships. Among 437 African immigrants, 60% were males, mean age was 47 years, 61% had lived in the U.S. for ≥10 years, mean length of stay was 15 years, and 81% were employed. Only 67% were insured. In the 12 months prior, 85% had screened for hypertension, 45% for diabetes, and 63% for dyslipidemia. African immigrants with a ≥10-year length of U.S. stay had 2.20 (95%Confidence Intervals: 1.31–3.67), and those with >25% years of life spent in the U.S. had 3.62 (95%CI: 1.96–6.68) higher odds of dyslipidemia screening compared to those with a <10-year length of stay and ≤25% years of life spent in the U.S., respectively. Overall, screening for CV risk higher in African immigrants who have lived longer (≥10 years) in the U.S. Recent African immigrants may experience challenges in accessing healthcare. Health policies targeting recent and uninsured African immigrants may improve access to CV screening services.

## 1. Introduction

Cardiovascular disease (CVD) remains a leading cause of death in the United States (U.S.) and globally [1] Although CVD risk factors, such as hypertension and diabetes, are modifiable through lifestyle changes, the burden of CVD remains high and accounts for one-third of deaths in the U.S. every year [2]. Screening is a proven primary prevention strategy to detect underlying risk factors and identify persons at risk for CVD events to prevent future complications [3]. For instance, The U.S. Preventive Services Task Force recommends yearly screening for adults age 40 years or older and those at increased risk for hypertension [4]. Black adults in the U.S. have a higher prevalence of hypertension and diabetes and higher rates of CVD mortality than other racial/ethnic groups [1]. 

African immigrants are a fast-growing immigrant population and subgroup of Black adults in the U.S. [5]. Over the last four decades, the number of African immigrants has increased from 130,000 to over 2 million [5]. Although research among African immigrants in the U.S. is limited, a few studies have shown that African immigrants may have better cardiometabolic health but lower access to healthcare than African Americans [6]. The “healthy immigrant effect,” a phenomenon where immigrants tend to be healthier than the host population when they first arrive in the host country [7,8,9], has been proposed as an explanation for the apparent health advantage among immigrants. However, emerging evidence suggests that the healthy immigrant effect may diminish with the increasing length of residency due to lifestyle changes associated with acculturation [10,11]. 

Acculturation may help describe how African culture may impact health behaviors, including cardiovascular risk screening. Acculturation is defined as “the dual process of cultural and psychological change that occurs as a result of contact between two or more cultures and their individual members” [12,13]. One of the proxy measures of acculturation, such as length of stay in the U.S., is associated with increased prevalence of cardiometabolic factors among immigrants [14,15,16]. A study of acculturation and CVD risk in African immigrants from Ghana and Nigeria suggested that longer residence in the U.S. elevated CVD risks by increasing overweight/obesity and hypertension [17]. After migration, socioeconomic challenges, including poor education, income, and lack of health insurance, can increase risk for CVD among immigrants [18]. Although it is known that acculturation affects immigrant health, little evidence exists in the relationship between acculturation and CVD risk screening among African immigrants. 

Considering the rapid growth of the African immigrant population in the U.S. and their CVD risk, it is important to examine cardiovascular screening in this population. Therefore, the objective of this study was to examine the association between length of stay (a proxy for acculturation) and percent of life spent in the U.S. and cardiovascular disease risk screening among African immigrants in the U.S. 

## 2. Materials and Methods

### 2.1. Participants

The African Immigrant Health Study (AIHS) was a cross-sectional study of first-generation African immigrants from Ghana, Nigeria, Liberia, Sierra Leone, and Cameroon, living in the Baltimore–Washington D.C. metropolitan area [19]. Using convenience sampling, participants were recruited from community-based organizations and religious institutions. Many Africans identify with faith-based organizations and places of worship, such as churches and mosques, as these organizations provide immigrants and minority ethnic groups with a supportive and trusting environment [20]. Recruiting from religious organizations has also been successfully employed when recruiting “hard-to-reach” populations, such as African immigrants [20]. The inclusion criteria for the AIHS study included being 30 years or older and born in one of the following countries: Liberia, Ghana, Cameroon, Nigeria, or Sierra Leone; able to communicate in English; and living in the Baltimore–Washington D.C. metropolitan area. Participants born in the U.S. or pregnant women were excluded from the study. The study was approved by the Johns Hopkins School of Medicine Institutional Review Board Institutional Review Board. 

Data collection took place between June 2017 and April 2019; participants completed study surveys. The survey was self-administered and contained questions on sociodemographic characteristics, healthcare utilization behaviors, health history, screening behaviors, and psychosocial factors, such as social support, resilience, stress, and discrimination. In addition, blood pressure and anthropometric measurements were done by study staff.

### 2.2. Outcomes

The outcomes of interest were routine screening for the following CVD risk factors: hypertension, diabetes, and high cholesterol levels. Screening for the CVD risk factors was assessed with the following questions for hypertension: “During the past 12 months, have you had your blood pressure checked by a doctor, nurse, or other health professionals?”; for diabetes: “During the past 12 months, have you had a fasting test for high blood sugar or diabetes?”; and for high cholesterol: “During the past 12 months, have you had your blood cholesterol checked by a doctor, nurse, or other health professionals?” Responses to each of these were dichotomized as “Yes” or “No.”

### 2.3. Exposure

The exposure of interest in this study was acculturation. We examined length of U.S. residence, a proxy measure for acculturation as an exposure. In the survey, participants were asked how long they had lived in the U.S. This variable was categorized as <10 years or ≥10 years of U.S. stay. Percent of life spent in the United States was also examined as an exposure. This was calculated by dividing the length of U.S. residence by age at the time of the study [21]. 

### 2.4. Covariates

Covariates examined included age, education, income, health insurance, and employment. Age in years was examined as a continuous variable. Sex was dichotomized as “male” or “female.” Educational status was categorized as high school or graduate equivalency degree or less, some college, bachelor’s degree, and graduate degree. This variable was further dichotomized into “less than a bachelor’s degree” and “more than a bachelor’s degree.” Annual household income was recoded into the following categories: ≤USD 39,999, USD 40,000–USD 69,999, USD 70,000–USD 99,999, and ≥USD 100,000. Health insurance status was assessed with the question, “Do you currently have any kind of health coverage, including health insurance; prepaid plans, such as health management organizations; or government plans, such as Medicare/Medicaid?” Responses to this question were dichotomized as “Yes” or “No.” Employment status was also categorized as “employed” and “not employed.”

### 2.5. Statistical Analyses

The sociodemographic characteristics of the sample were stratified by length of stay, using means (±SD) or median (Interquartile Range) for continuous variables and proportions and percentages for categorical variables. We used *t*-tests for differences in means and chi-square test statistics for differences in proportions. We recruited 465 African immigrants into this study; after exclusion due to missing data, 437 participants were included in this analysis. Independent samples Student’s *t*-test and chi-square test were used to compare differences in cardiovascular risk screening between length of stay in the U.S. and percent of life spent in the U.S.

To examine the relationship between length of U.S. stay and cardiovascular risk screening, we conducted a multivariable logistic regression adjusting for age, sex, education, income, health insurance, and employment. Analyses for blood pressure, fasting blood glucose, and lipids screening were conducted separately. We also conducted a similar analysis to examine the relationship between percent of life spent in the U.S. and acculturation strategy and cardiovascular risk screening. The models were adjusted for age, sex, income, health insurance, education, and employment. We also examined interactions between age categories and sex and between age categories and length of stay and presented the adjusted predicted probabilities in plots. We summarized the prevalence of blood pressure, fasting blood glucose, and lipids screenings in graphs, including the percent difference by length of stay (<10 years compared to ≥10 years) and percent of life spent in the U.S. (≤25% compared to >25%). We examined temporal trends of screening rates by length of stay divided into four timepoints (<5, 5–9, 10–14, and ≥15 years). Analyses were performed with Stata/IC 16.1 [22]. A two-sided significance level of α < 0.05 for statistical significance was applied. 

## 3. Results

Table 1 provides details on the sample sociodemographic characteristics: 59.7% (261) were females, and mean age (±SD) was 46.9 (±11.6) years. Most participants were born in Ghana (156, 35.7%) and Nigeria (158, 36.2%). Mean BMI (±SD) was 30.3 (±6.3); 32.5% (142) were overweight, and 53.1% (232) were obese. The mean length of stay in the U.S. was 15 years, and 61% had lived in the U.S. for ≥10 years. Compared to those with <10 years length of U.S. stay, African immigrants who had lived in the U.S. for ≥10 years had higher BMI than those with <10 years of residence. (29.0 vs. 30.6, *p* = 0.001). Similarly, persons who had spent >25% of their lives in the U.S. had higher BMI than those with ≤25% life spent in the U.S. (29.9 vs. 34.4, *p* < 0.001). Persons with ≥10 years were more likely to have a graduate degree compared to those with <10 years of U.S. stay (35.5%, 93 vs. 25.6%, 42, *p* = 0.001). Only 67.1% (288) had health insurance, and those with ≥10 years were more likely to have health insurance compared to those with <10 years (81.5% vs. 43.9%, *p* < 0.001). About 83.5% (360) were employed, and persons with ≥10 years of U.S. stay were also more likely to be employed (88.6% vs. 75.6%, *p* < 0.001).

### 3.1. Prevalence of Cardiovascular Screening

**Figure 1:**
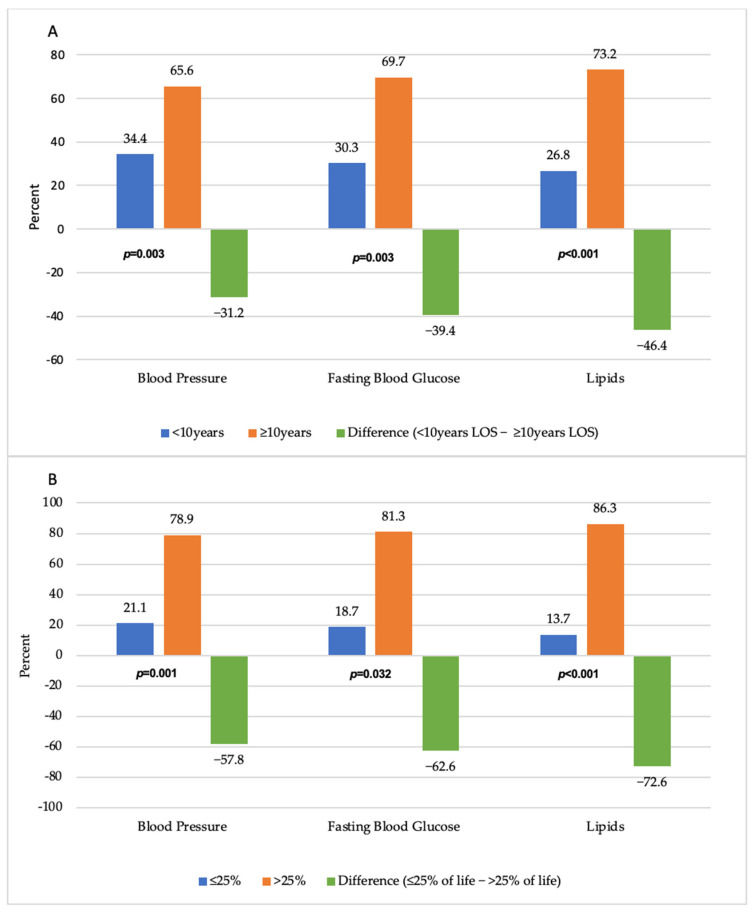
Prevalence of blood pressure, fasting blood glucose, and lipids screening by acculturation proxy ((**A**) length of stay (<10, ≥10 years) and (**B**) percent of life spent in the U.S. (≤25, >25 years)) and percent differences.

Overall, in the 12 months prior to data collection, 85.3% of participants were screened for hypertension, 45.0% were screened for diabetes, and 62.6% were screened for high cholesterol (Figure 1). We examined screening rates by length of U.S. stay. Among those with ≥10 years of U.S. stay, blood pressure (65.6% vs. 34.3%, *p* < 0.05; diff: −31.2%), fasting blood glucose (69.7% vs. 30.3%, *p* < 0.05; diff: −39.4%), and lipids (73.2% vs. 26.8%, *p* < 0.001; diff: −46.4%) screening rates were higher compared to those who have lived in the U.S. for less than 10 years. We noticed a similar trend in screening differences among those who had spent at least 25% of their lives in the U.S. in comparison to those with ≤25% of life spent in the U.S. for blood pressure (78.9% vs. 21.1%, *p* < 0.05; diff: −57.8%), fasting blood glucose (81.3% vs. 18.7%, *p* < 0.05; diff: −62.6%), and lipids (86.3% vs. 13.7%, *p* < 0.001; diff: −72.6%).

### 3.2. Differences in Screening Rates by Age, Sex, and Health Insurance Status

**Figure 2:**
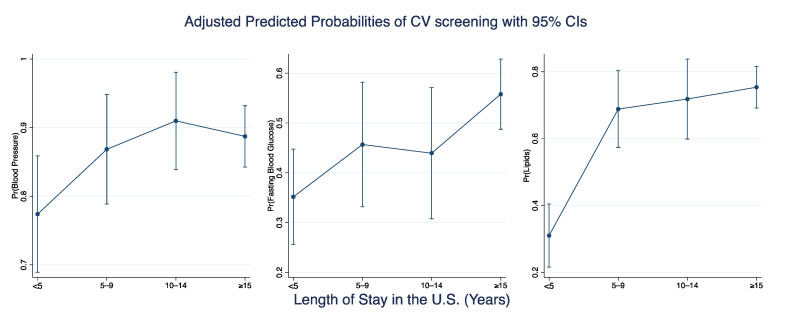
Adjusted predicted probabilities for CV screening (blood pressure, fasting blood glucose, and lipids).

**Figure A1:**
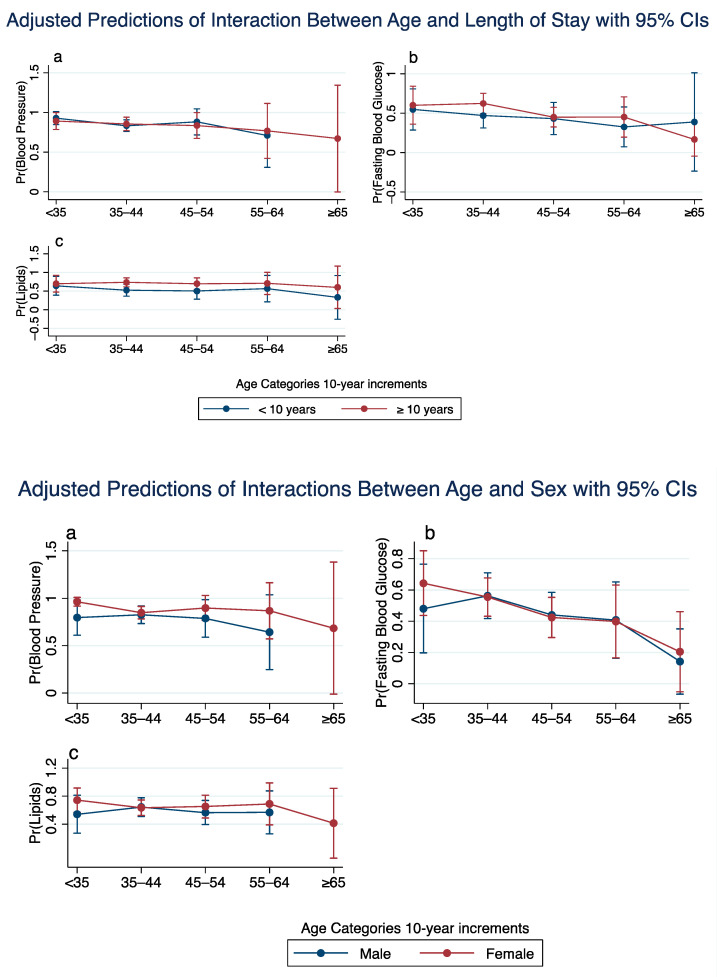
(**a**–**c**) Adjusted predictive probabilities for interactions between age and length of stay (**Top**) and between age and sex (**Bottom**).

We examined screening rates over length of stay divided into four timepoints, namely <5 years, 5–9 years, 10–14 years, and ≥15 years (Figure 2). Screening rates for blood pressure, fasting blood glucose, and lipids increased steeply between <5 years to 5–9 years of U.S. stay. However, the differences were tapered over the subsequent three time points, and for blood pressure, we observed a downward trend after 10-year stay in the U.S. In both interactions between age categories and sex and between age categories and length of stay, we did not observe a significant separation on the curves (Appendix A, Figure A1a,b). However, for each age category, females and those with ≥10 years length of stay were likely to consistently have higher screening rates. We also observed differences in screening rates by health insurance status; persons who have health insurance were more likely to report CV screening (Appendix A, Table A1). Among persons who have health insurance compared to those without, the prevalence of blood pressure screening was 90.8% vs. 73.4%; for fasting blood glucose, 50.9% vs. 33.3%; and for lipids, 72.7% vs. 42.7%. In the multivariable logistic regression models, persons with health insurance had 2.89 higher odds of screening for hypertension compared to those with no health insurance (adjusted odds ratio (aOR): 2.89, 95%CI: 1.51–5.56); finding were similar for diabetes screening (aOR: 2.21, 95%CI:1.37–3.58) and for dyslipidemia screening (aOR: 3.57, 95%CI: 2.15–5.92).

### 3.3. Cardiovascular Disease Screening

In the past 12 months, 85% of the participants were screened for hypertension, 45% for diabetes, and 63% for dyslipidemia. After adjusting for covariates, African immigrants with ≥10-year length of U.S. stay had 2.20 (adjusted odds ratio (OR): 2.20, 95% Confidence Intervals (CI): 1.31–3.67) higher odds of being screened for dyslipidemia in the past 12 months compared to those with <10-year length of stay (Table 2). For diabetes, those who have lived in the U.S. for ≥10 years had 1.43 (adjusted OR: 1.43, 95%CI: 0.88–2.35) higher odds of being screened for diabetes. However, this was not statistically significant.

For percent of life spent in the U.S., 25.1% (107) of the participants have spent at most 25% of their lives in the U.S., while 74.9% (319) have spent more than 25% of their lives in the U.S. After adjusting for covariates, African immigrants who have spent >25% of their lives in the U.S. had 3.62 (adjusted odds ratio: 3.62, 95%CI: 1.96–6.68) higher odds of being screened for dyslipidemia in the past 12 months compared to those with ≤25% of their lives spent in the U.S. (Table 3).

### 3.4. Sociodemographic Determinants of Cardiovascular Screening

We also examined the social determinants of CV screening (Table A2). Per-year increase in age was associated with higher odds of screening for blood pressure (aOR: 1.25, 95%CI: 1.02–1.10); fasting blood sugar (aOR: 1.03, 95%CI: 1.01–1.06); and lipids screening (aOR: 1.07, 95%CI: 1.04–1.10). Having health insurance was associated with higher odds of CV screening for blood pressure (aOR: 2.98, 95%CI: 1.41–6.27); fasting blood sugar (aOR: 2.08, 95%CI: 1.23–3.51); and lipids (aOR: 3.12, 95%CI: 1.81–5.40). Additionally, being female was associated with higher odds of blood pressure screening (aOR: 3.38, 95%CI: 1.69–6.76). Compared to African immigrants from Ghana, Nigerian and Liberian immigrants were less likely to be screened for diabetes (Nigerian immigrants, aOR: 0.44 95%CI: 0.25–0.76; Liberian immigrants, aOR: 0.40 95%CI: 0.19–0.83), and Sierra Leonean immigrants had a lower odds for blood pressure screening (aOR: 0.19 95%CI: 0.04–0.81), while Nigerian immigrants had a lower odds of lipid screening aOR: 0.47 95%CI: 0.26–0.84).

## 4. Discussion

We sought to examine the association between acculturation, measured by length of stay and percent of life spent in the U.S., and cardiovascular risk screening among African immigrants in the U.S. Among African immigrants residing in a large metropolitan area, we found that 85%, 45%, and 64% of participants reported screening for hypertension, diabetes, and high cholesterol, respectively, in the prior 12 months. In addition, African immigrants who had lived for 10 years or more were two times more likely to have been screened for high cholesterol in the past 12 months compared to those who had lived in the U.S. for less than 10 years. Our findings also showed that African immigrants who had spent 25% or more of their lives living in the U.S. were about 3.5 times more likely to have been screened for high cholesterol than those who had spent less than 25% of their lives in the country. Although the results for hypertension and diabetes were not statistically significant, we noticed a similar trend suggesting that longer U.S. stay may be associated with health care access, utilization, and preventive CV screening.

Our results corroborate previous studies, which have demonstrated that immigrants have lower preventive care screening rates than U.S.-born adults [2,23]. In a nationally representative study of U.S. immigrants, blood pressure screening, blood cholesterol screening, and blood sugar screening were higher among immigrants who had spent 15 years or more in the country [2]. Another study among Mexican immigrants found a persistent disparity in lipid screening for over 20 years [23]. We have further demonstrated that length of time spent in the U.S. is associated with preventive screening for high cholesterol. Although prior studies have shown differences in nativity status and lipid screening, our study is one of the first few studies showing differences in screening by acculturation (length of U.S. stay and percentage of life spent in the U.S.) among African immigrants. Studies have shown inadequate access to healthcare services, immigration status, language barriers, and lower socioeconomic status are important risk factors for lower screening for dyslipidemia [23,24,25].

Our findings also showed a lower health insurance rates among African immigrants, with only 67% of them reporting having health insurance. This is low compared to the national average, with 91.4% health insurance coverage [26]. Compared to other immigrant groups, African immigrants were the least likely to have health insurance, a primary care provider, or a usual place for healthcare [2]. Inadequate or lack of health insurance has been reported as a structural barrier to preventive health services, including CV screening [27]. In our study, we observed significant differences in CV screening rates by health insurance status; overall, persons who had health insurance had higher CV screening rates compared to those without health insurance. Difficulty accessing or navigating health insurance and high out-of-pocket cost for those without insurance are major barriers to utilizing preventive services [28,29]. Policies, such as the Personal Responsibility and Work Opportunity Reconciliation Act of 1996 that blocks immigrants’ access to government insurance programs until after 5 years of legal U.S. residence, are major exclusionary barriers. Such lengthy wait periods for qualifications for public assistance benefits and healthcare access and public charge rules are barriers that further impede health insurance coverage among African immigrants and contribute to worse health outcomes [30,31,32]. Policies of inclusion that provide immigrants with access to health insurance and health care would be highly beneficial to the United States population and economy, facilitating mutual understanding and trust and better health outcomes [31].

Current evidence has shown mixed findings on cardiovascular risk screening among immigrant populations. For instance, a population-based study showed that the immigrant group had a higher rate of diabetes screening in comparison to the general population group (76.0% vs. 74.4%, *p* < 0.001), with the highest rates in people originating from South Asia, Mexico, Latin American, and the Caribbean [33]. Yet, an analysis of the 1988–2008 National Health and Nutrition Examination Surveys demonstrated that individuals born in Mexico were less likely to engage in cholesterol screening than the U.S. natives or U.S.-born Hispanic persons (70.9%, 80.1%, 77.8%, respectively) [23]. Those conflicting results may be explained by the difference in the immigrants’ region of origin and their cultural backgrounds [2]. Many African immigrants migrate from countries with limited healthcare resources, tepid attitudes towards preventive screening, lower screening rates, and low awareness of cardiometabolic diseases [34]. Hence, African immigrants’ health-seeking behavior may not be aligned with the U.S. context, and they are likely to not prioritize preventive screenings. Cultural beliefs and attitudes towards preventative healthcare, religious beliefs/views, and mistrust of Westernized healthcare influence preventative health-seeking behavior among African immigrants [35]. Multi-level interventions are necessary to improve preventive CV screening in this population.

CVD risk in immigrants often mirrors the risk of the host population with increased length of stay in the host country [36]. Studies have shown that immigrants are more likely to underutilize preventive CVD screening compared to U.S.-born persons [2,23]. Indeed, previous studies reported a higher prevalence of hypertension, obesity, diabetes, and high cholesterol among African immigrants compared with their U.S.-born counterparts [37]. Preventive screening for CVD helps with early diagnoses and interventions. Unfortunately, many CVD and CVD risk factors have ominous symptoms and remain undiagnosed [2]. The American College of Cardiology and American Heart Association (ACC/AHA) recommend lipid profile testing for persons 20 years and older [38]. Hence, given the rapid increase in the African immigrant population in the U.S. and the rates of CVD mortality among Black people, it is critical that preventive care is provided to this population. Routine and timely screening can help healthcare providers identify CVD risk factors and prevent this population from developing CVD.

This study has some limitations, which must be considered. First, the cross-sectional design of this study limits our ability to establish temporality or causation. Second, our findings may be subject to recall bias, as the outcomes were based on self-report of screening in the prior 12 months, which may either underestimate or overestimate the prevalence of CV screening. Third, we employed convenience sampling and recruited participants from faith-based organizations, which may introduce social desirability bias and limit our findings’ generalizability. Despite these limitations, this study has some major strengths. First, to our knowledge, this is one of the first few studies to examine the association between acculturation and CV screening among African immigrants. This finding is a significant contribution to the literature, providing an opportunity to understand the CV screening behavior and health needs of this population. Second, our study recruited a relatively homogeneous sample of West African immigrants who share similar geopolitical histories and provided an adequate representation of women. Third, using community-engaged approaches, this study was successful in recruiting African Immigrants who have been described as a hard-to-reach population [39].

## 5. Conclusions

Preventive screening is essential for early detection and early intervention, including minimizing complications from CVD and other related chronic diseases. Recent African immigrants to the U.S. may experience challenges in accessing healthcare. To mitigate these challenges, health policies should be developed that facilitate early and better integration of immigrants into the health care system. Community-based screening events and linkage to care (i.e., connecting African immigrants to health and medical care services) may improve the CV health of African immigrants. 

## Figures and Tables

**Table 1:** Sociodemographic characteristics of African Immigrants stratified by length of U.S. stay.

Characteristics, M (±SD)/*n* (%)	Total	Length of Stay		
		<10 Years	≥10 Years	
	*n* = 437	*n* = 170	*n* = 267	*p*-Value
Age, years, M (±SD)	46.9 (11.6)	42.9 (10.7)	49.0 (11.5)	<0.001 *
Sex, *n* (%)				0.93
Male	176 (40.3)	68 (40)	108 (40)	
Female	261 (59.7)	102 (60)	159 (60)	
BMI, kg/m^2^, M (±SD)	30.3 (6.3)	29.0 (4.8)	30.6 (5.0)	0.001 *
BMI categories (kg/m^2^), *n* (%)				0.001 *
Normal weight (18.5–24.9)	62 (14.2)	35 (20.6)	27 (10.1)	
Overweight (25.0–29.9)	142 (32.5)	63 (37.1)	79 (29.6)	
Obese (≥30)	232 (53.1)	72 (42.4)	160 (59.9)	
Country of origin, *n* (%)				<0.001 *
Ghana	156 (35.7)	38 (22.4)	118 (44.2)	
Nigeria	158 (36.2)	103(60.6)	55 (20.6)	
Liberia	53 (12.1)	5 (2.9)	48 (18.0)	
Sierra Leone	21 (4.8)	2 (12.9)	27 (10.11)	
Cameroon	49 (11.2)	22 (12.9)	27 (10.1)	
Educational status, *n* (%)				0.001 *
High School diploma/≤GED	11 (2.6)	6 (3.7)	5 (1.9)	
Some college	140 (32.9)	45 (27.4)	95 (36.3)	
Bachelor’s degree	140 (32.9)	71 (43.3)	69 (26.3)	
Graduate degree	135 (31.7)	42 (25.6)	93 (35.5)	
Household Income, *n* (%)				<0.001 *
≤USD 39,999	74 (18.1)	47 (30.5)	27 (10.6)	
USD 40,000–USD 69,999	101 (24.8)	37 (24.0)	64 (25.2)	
USD 70,000–USD 99,999	102 (25.0)	27 (17.5)	75 (29.5)	
≥USD 100,000	131 (32.1)	43 (27.9)	88 (34.7)	
Health insurance, *n* (%)				<0.001 *
No	141 (32.9)	92 (56.1)	49 (18.5)	
Yes	288 (67.1)	72 (43.9)	216 (81.5)	
Employment status, *n* (%)				<0.001 *
No	71 (16.5)	41 (24.4)	30 (11.4)	
Yes	360 (83.5)	127 (75.6)	233 (88.6)	
Marital status, *n* (%)				0.93
Not married/cohabiting	130 (29.8)	50 (29.6)	80 (30.0)	
Married/cohabiting	306 (70.2)	119 (70.4)	187 (70.0)	
Social Support, *n* (%)				0.31
Low	280 (64.1)	104 (61.2)	176 (65.9)	
High	157 (35.9)	66 (38.8)	91 (34.1)	
Migration Reasons, *n* (%)				0.378
Education	103 (24.9)	31 (19.1)	72 (28.7)	
Economic hardship	28 (6.8)	13 (8.0)	15 (6.0)	
To join family	136 (32.9)	57 (35.2)	79 (31.5)	
Asylum/Refugee	36 (8.7)	14 (8.6)	22 (8.8)	
Job opportunities	54 (13.1)	24 (14.8)	30 (12.0)	
Other reasons	56 (13.6)	23 (14.2)	33 (13.2)	
Percentage of life in the U.S., *n* (%)				<0.001 *
≤25%	107 (25.1)	106 (63.9)	1 (0.4)	
>25%	319 (74.9)	60 (36.1)	259 (99.6)	

* Indicates statistically significant values, *p* <  0.05. *p*-values estimated using *t*-tests for differences in means and chi-square test statistics for differences in proportions. BMI, body mass index; M, mean; SD, standard deviation; GED, general educational development diploma; kg/m^2^, kilograms/meters^2^.

**Table 2:** Association between cardiovascular screening and length of residence (*n* = 437).

	Length of Stay			
	≥10 Years ^a^		≥10 Years ^a^	
Self-Reported CV Screening	Unadjusted		Adjusted ^b^	
	OR	95% CI	AOR ^b^	95% CI
Hypertension	2.36	1.38–4.06 *	1.00	0.50–2.00
Diabetes	1.97	1.32–2.93 *	1.43	0.88–2.35
Dyslipidemia	3.99	2.63–6.06 *	2.20	1.31–3.67 *

* Indicates statistically significant values, *p*  <  0.05. OR, odds ratio; AOR, adjusted odds ratio; CI, confidence interval; CV, cardiovascular. ^a^ Reference group: length of U.S. stay <10 years. ^b^ Adjusted for age, sex, education, income, insurance, and employment.

**Table 3:** Association between cardiovascular screening and percent of life spent in the U.S. (*n*  =  426).

	Percent of Life Spent in the U.S.			
	>25% ^a^		>25% ^a^	
Self-Reported CV Screening	Unadjusted		Adjusted ^b^	
	OR	95%CI	AOR	95%CI
Hypertension	2.56	1.45–4.54 *	1.16	0.53–2.51
Diabetes	1.75	1.11–2.77 *	1.11	0.62–2.00
Dyslipidemia	4.90	3.05–7.89 *	3.62	1.96–6.68 *

* Indicates statistically significant values, *p* < 0.05. OR, odds ratio; AOR, adjusted odds ratio; CI, confidence interval; CV, cardiovascular; ^a^ Reference group: ≤25 percent of life spent in the U.S. ^b^ Adjusted for age, sex, education, income, insurance, and employment.

## Data Availability

The data presented in this study are available in the articles and Appendix A.

## Appendix A

**Table A1:** Association between cardiovascular screening and health insurance status (*n*  =  437).

	Health Insurance Status					
			Insured ^a^		Insured ^a^	
	Insured	Not Insured	Unadjusted		Adjusted ^b^	
Self-Reported CV Screening	Prev, *n* (%)		OR	95% CI	AOR ^b^	95% CI
Hypertension	266 (90.8)	105 (73.4)	3.58	2.07–6.21 *	2.89	1.51–5.56 *
Diabetes	148 (50.9)	48 (33.3)	2.14	1.42–3.23 *	2.21	1.37–3.58 *
Dyslipidemia	210 (72.7)	61 (42.7)	3.80	2.49–5.80 *	3.57	2.15–5.92 *

* Indicates statistically significant values *p*  <  0.05. OR, odds ratio; AOR, adjusted odds ratio; CI, confidence interval; CV, cardiovascular. ^a^ Reference group: No health insurance. ^b^ Adjusted for age, sex, education, income, and employment.

**Table A2:** Sociodemographic Determinants of Cardiovascular Screening in the Past 12 Months Among African Immigrants (*n* = 437).

Self-Reported CV Screening	Hypertension	Diabetes	Dyslipidemia
	aOR (95% CI)	aOR (95% CI)	aOR (95% CI)
Age (per year)	1.25 (1.02–1.10) *	1.03 (1.01–1.06) *	1.07 (1.04–1.10) *
Female (Ref.: Male)	3.38 (1.69–6.76) *	1.34 (0.86–2.11)	1.58 (0.96–2.61)
Employed (Ref.: Not employed)	1.88 (0.75–4.73)	0.86 (0.44–1.68)	2.00 (0.94–4.18)
≥Bachelor’s degree (Ref.: < Bachelor’s degree)	0.75 (0.33–1.69)	1.30 (0.77–2.18)	1.40 (0.79–2.49)
Insured (Ref.: No Health Insurance)	2.98 (1.41–6.27) *	2.08 (1.23–3.51) *	3.12 (1.81–5.40) *
Household Income (Ref.: ≤ USD 39,999)			
USD 40,000–USD 69,999	1.10 (0.42–2.92)	0.56 (0.28–1.12)	1.17 (0.55–2.45)
USD 70,000–USD 99,999	0.99 (0.35–2.78)	0.90 (0.45–1.83)	1.58 (0.73–3.42)
≥ USD 100,000	1.12 (0.44–2.86)	0.69 (0.35–1.36)	1.14 (0.55–2.37)
Country of origin (Ref.: Ghana)			
Nigeria	0.54 (0.24–1.23)	0.44 (0.25–0.76) *	0.47 (0.26–0.84) *
Liberia	1.26 (0.25–6.28)	0.40 (0.19–0.83) *	0.58 (0.24–1.40)
Sierra Leone	0.19 (0.04–0.81) *	0.40 (0.14–1.19)	1.29 (0.30–5.60)
Cameroon	1.24 (0.35–4.40)	0.48 (0.23–1.00)	0.49 (0.22–1.10)
Traditionalist acculturation style (Ref.: Integrationist)	1.25 (0.38–4.04)	1.11 (0.55–2.25)	1.48 (0.38–5.74)

* Indicates statistically significant values *p*  <  0.05. aOR, adjusted odds ratio; CI, confidence interval; CV, cardiovascular. Adjusted OR indicates that the variable was adjusted for all other sociodemographic characteristics in the table specifically Adjusted for age, sex, employment, education, health insurance, income, country of origin, and acculturation strategy.
